# Triggers, Risk Factors, and the Prevalence of Syncope Among Domestic Hajj Pilgrims, 2023: A Cross-Sectional Study

**DOI:** 10.7759/cureus.62201

**Published:** 2024-06-11

**Authors:** Rehab A Mohammed, Intessar Sultan, Abdulrahman A Shamakh, Adnan A Balamesh, Ahmed J Kishta, Loai A Alkhotani

**Affiliations:** 1 Internal Medicine, Ibn Sina National College for Medical Studies, Jeddah, SAU; 2 Internal Medicine, Al-Azhar University Faculty of Medicine for Girls, Cairo, EGY; 3 Medical School, Ibn Sina National College for Medical Studies, Jeddah, SAU

**Keywords:** pilgrimage, hajj, loss of consciousness, lightheadedness, collapse, fainting, syncope

## Abstract

Background: Syncope and other transient loss of consciousness episodes in crowded and unfamiliar environments may lead to major health hazards. Despite numerous publications, data on syncope among Hajj pilgrims in Makkah is lacking.

Objectives: To identify the triggers, risk factors, and prevalence of syncope and other transient loss of consciousness episodes among domestic pilgrims.

Methodology: This cross-sectional study included a convenient sample of domestic pilgrims who performed Hajj in July 2023 using an online Google Forms questionnaire (Alphabet Inc., Mountain View, CA).

Results: Out of 388 participants, 69 (18.1%) reported a history of syncope during the Hajj pilgrimage. Among these, 57 (82.6%) reported complete loss of consciousness, and 56 (81.2%) noted warning symptoms preceding the episode. The syncopal attack occurred once in 49 respondents (71%). Several triggers for syncope were identified, with sudden standing from a sitting position being the most prevalent (100%). Additional co-triggers were crowding (n=43; 62.3%), stressful conditions (n=30; 43.2%), prolonged standing (n=21; 30.4%), and walking (n=11; 15.9%). Traumatic injuries were reported in 33 (47.8%) as a result of syncope. Standing for long periods of time on the day of Arafat (Arafat standing) emerged as the most common triggering situation (n=48; 69.6%). There were multiple medical factors contributing to syncopal episodes; the most common medical explanations were heat exhaustion (n=48; 69.6%), dehydration (n=24; 34.8%), over-exertion (n=48; 69.6%), low blood sugar (n=10; 14.5%), and low blood pressure (n=17; 24.6%). Significant predictors were the presence of cardiac disease (odd ratio (OR) 7.6, 95% confidence interval (CI) 2.71-21.45, p<0.001), anemia (OR 2.5, 95% CI 1.01-6.09, p=0.049), previous syncope (OR 2.5, 95% CI 1.02-6.27, p=0.049, and family history of syncope (OR 10.1, 95% CI 2.08-49.32, p=0.004).

Conclusion: Syncope during the domestic Hajj pilgrimage is frequent, especially on the day of Arafat, and carries the risk of traumatic injury. People with previous episodes of syncope and comorbidities, especially cardiac patients and those who have a family history of syncope, are particularly prone to this risk. Healthcare should focus on at-risk patients, particularly on critical pilgrimage days, and increase pilgrims' awareness about triggers of syncope including sudden and prolonged standing, exertion, and heat exposure.

## Introduction

The Hajj pilgrimage is one of the largest religious journeys in the world, presenting unique public health challenges. The season of the Hajj varies due to its adherence to the Islamic lunar calendar. The Hajj takes place in month 12 of the lunar calendar for five days, from 8 to 12 [1]. 

Hajj rites are grouped into Tawaf (circumambulation of Kaaba, an aggregate distance of about 40 km), a ritual of Saee (running seven times between two small hills, Safa and Marwah), the day of Arafat (8 miles east of Makkah, a highlight of the Hajj), spending the night at Muzdalifa, stoning the Jamarat (the densest crowds during the Hajj), animal sacrifice, staying in Mina, and culminating with the final farewell ritual of Tawaf and Saee [1].

In 2023, the Hajj took place in July, a summer month. Therefore, the pilgrims were exposed to diverse and significant health risks due to the excessive heat, limited time, the confined geographical area of the event, and the large numbers of people, amounting to millions of people [2]. Over decades, various health risks at the Hajj were reported, affecting even domestic pilgrims residing in the Kingdom of Saudi Arabia (KSA). For decades, various health risks have been documented during the Hajj, including among domestic pilgrims residing in the Kingdom. The healthcare system has implemented rigorous preparations to monitor the vast numbers of pilgrims in a relatively confined space considering diverse cultural, linguistic, and, most significantly, medical backgrounds [1,3].

Syncope, a sudden loss of consciousness associated with the inability to maintain a postural tone, followed by spontaneous recovery, is relatively common. Although syncope has many possible causes, the cardiac cause is the most serious category and may be linked to increased mortality. Patients with cardiac syncope constitute a high-risk group predisposed to morbidity and premature mortality from cardiovascular disease and should be monitored closely. Even for those with unknown cause for syncope, it appears that they are at an increased risk for death. Moreover, there is an increased risk of stroke among those with underlying neurologic causes for their syncope [4]. Additionally, syncope can lead to serious traumatic injuries in case the patient falls in crowded or unfamiliar environments.

The prevalence of syncope among pilgrims could vary according to destination, length of their journey, pilgrim's age, and health status. Many risk factors can provoke it, including low blood pressure, dehydration, heat illnesses, heart problems, and other complications. Triggers of syncope during the Hajj include high environmental temperature, prolonged standing and walking, psychological stress, or sudden changes in posture [4,5]. Previous studies have investigated different aspects of health risks (2), including cardiovascular diseases [5] and heat-related illnesses [6] during pilgrimages. However, separate studies are lacking in the literature regarding syncope and other transient loss of consciousness such as seizures, especially generalized tonic-clonic seizures, and metabolic disturbances conditions such as hyperventilation and electrolyte imbalances. 

Identifying the triggers and risk factors associated with syncope during pilgrimages is important for developing effective preventive measures and improving the safety of pilgrims during their journey. Therefore, this study was conducted to identify the triggers, risk factors, and prevalence of syncope and other transient loss of consciousness episodes among domestic pilgrims during the 2023 Hajj period.

## Materials and methods

The study design is a cross-sectional population-based survey that included male and female resident pilgrims of all ages and nationalities who undertook the pilgrimage from inside KSA in July 2023, considered to be the hottest month of the year in the region. The required sample size was estimated using the Qualtrics calculator (Qualtrics International Inc., Provo, UT; Seattle, WA) at a 95% confidence level, a margin of error of ±5%, and an assumed prevalence of 50%. The required minimum sample size was determined to be 385. Sampling was performed using a non-randomized, convenient, consecutive technique. Data was collected using an online multiple-choice, anonymous questionnaire distributed through social media portals of internal pilgrims' groups using Google Forms (Alphabet Inc., Mountain View, CA). The questionnaire included questions about demographic data, risk factors of syncope, details of syncopal events, triggering factors, and medical consultation. The questionnaire was constructed based on the literature review by an expert (consultant internist) and reviewed by three other consultants (one family medicine consultant and two consultant internists) for validity.

Ethical consideration

A detailed online informed consent was obtained from each participant before replying to the questionnaire. All data involved in the questionnaire were used for the sole purposes of this research. The study was conducted after the approval of the Institutional Research Review Board at Ibn Sina National College ISNC (IRRB-01-17092023).

Statistical analysis

Statistical data was analyzed using SPSS software (version 22.0; SPSS Inc., IBM Corp., NY). Data were reported as the number and the frequency of categorical variables. Significant risk factors and triggers were identified using binary regression analysis. Binary regression analysis included all demographic data, chronic illness, family, and past history for the model prediction with odd ratio (OR) and 95% confidence interval estimation for significant predictors. A two-sided P-value <0.05 was considered the level of significance for all tests. Binary regression analysis showed that the chi-square model was 75.97, df 18, p <0.001 with a Nagelkerke R square of 29.3%. The model was well-fitting with the goodness of fit test (Hosmer and Lemeshow test was > 0.05 p=0.723). 

## Results

The study included 248 (63.9%) Saudi and 140 (36.1%) non-Saudi participants. Males accounted for 51% (n=198) and females 49% (n=190) with different age groups, with only 1.37% (n=5) in the elderly age group. The most commonly reported chronic illnesses among participants were obesity (n=104; 26.8%) and diabetes (n=99; 25.5%), followed by hypertension, (n=62; 16%), hypotension (n=57; 14.7%), and anemia (n=51; 13.1%). A history of cardiac disease was reported in 37 (9.5%), and a previous syncope in 66 (17%) (Table 1).

**Table 1:** Demographic characteristics of the participants Data is represented as numbers (n) and percentages (%)

Characteristics		Participants (n=388)	
		n	%
Gender	Male	198	51.00%
	Female	190	49.00%
Age	18-24	27	7%
	25-34	72	18.70%
	35-44	121	31.30%
	45-54	118	30.50%
	55-64	45	11.70%
	>65	5	1.40%
Nationality	Saudi	248	63.90%
	Non-Saudi	140	36.10%
Occupation	Employed	172	44.30%
	part-time	27	7.00%
	self-employed	68	17.50%
	Unemployed	14	3.60%
	Student	19	4.90%
	Retired	30	7.70%
	Housewife	58	14.90%
Chronic illnesses	Obesity	104	
	Hypertension	62	
	Diabetes	99	
	Hyperlipidemia	61	
	Cardiac disease	37	
	Hypotension	57	
	Anemia	51	
History of syncope before pilgrimage		66	

**Figure 1:**
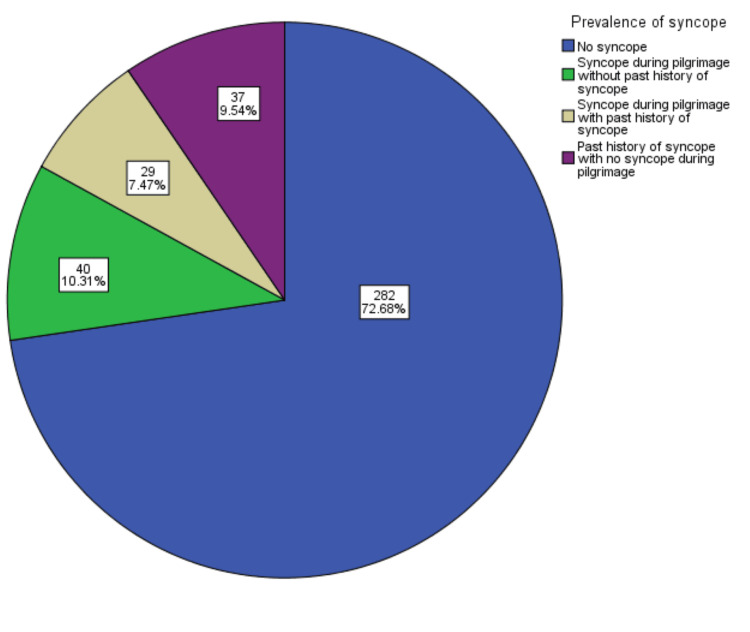
Prevalence of syncope during performing domestic pilgrimage

During the pilgrimage, 69 (18.1%) out of 388 participants reported a history of syncope, with 40 (10.3%) experiencing their first syncopal episode. In comparison, 37 (9.54%) had a previous history but did not suffer from syncope during the Hajj period (Figure 1).

**Figure 2:**
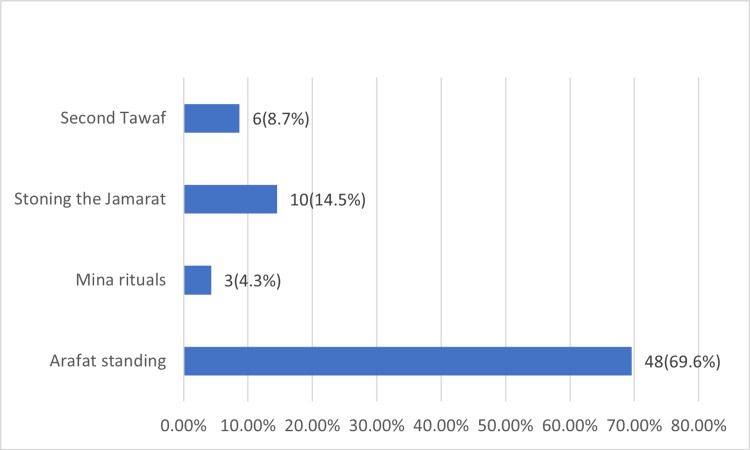
Syncope at Hajj pilgrimage rituals

Among those who experienced syncope during the pilgrimage, 49 (71%) reported it only once, while 20 (29%) experienced it more than once. A total of 57 participants (82.6%) reported complete loss of consciousness during their syncopal episode, 17 (24.5%) reported very brief loss of consciousness <1 min, and very few (n=2; 2.9%) reported longer duration up to 10 min. On the other hand, a considerable portion of 42 (60.9%) participants could not accurately estimate their syncope duration. A total of 56 (81.2%) participants suffered from syncope without preceding warning symptoms. All syncopal attacks (100%) were reported after suddenly standing from a sitting position. Additional co-triggers of syncope were crowding (n=43; 62.3%), stressful conditions (n=30; 43.2%), prolonged standing (n=21; 30.4%), and walking (n=11; 15.9%). Unfortunately, 33 (47.8%) of the pilgrims suffered injuries as a result of syncope. Most syncopal attacks occurred during the day of Arafat, or Arafat standing; n=48; 69.6%) followed by stoning the Jamarat (n=10; (14.49%), and "Tawaf Al-Ifadah", or second Tawaf (n=6; 8.7%) (Figure 2).

While a majority of participants (n=56; 81.2%) sought medical consultation for their syncope episodes, a notable proportion (n=13; 18.8%) did not. Multiple medical explanations were provided with the most common explanations being heat exhaustion (n=48; 69.6%), dehydration (n=24; 34.8%), over-exertion (n=48; 69.6%), low blood sugar (n=17; 14.5%), low blood pressure (n=10; 24.6%), and lastly, stress (n=3; 4.3%) (Table 2).

**Table 2:** Characteristics of syncope during performing domestic pilgrimage Data is represented as numbers (n) and percentages (%)

Parameters		Syncope during pilgrimage (n= 69;18.1%)	
		n	%
Frequency	Once	49	71.00%
	More than once	20	29.00%
Preceding warning symptoms (lightheadedness, nausea, or dizziness)	Yes	56	81%
	No	13	18.80%
Complete loss of consciousness	Yes	57	82.60%
	No	12	17.40%
Duration	< 1 minute	17	24.60%
	1-5 minutes	8	11.60%
	6-10 minutes	2	2.90%
	I don't know	42	60.90%
Triggers	Sudden standing from sitting position	69	100.00%
	During crowding	43	62.30%
	With stressful conditions	30	43.50%
	Prolonged standing	21	30.40%
	During walking	11	15.90%
Traumatic injuries	Yes	33	47.80%
	No	36	52.20%
Medical consultation	Yes	56	81.20%
	No	13	18.80%
Medical reasons	Heat illness	48	69.60%
	Dehydration	24	34.80%
	Over exertion	48	69.60%
	Low blood pressure	17	24.60%
	Low blood sugar	10	14.50%
	Stress	3	4.30%

Binary regression analysis showed that all demographic data, chronic illness, family, and past history represented a significant well-fitting model predicting syncope (chi-square of 75.97, df 18, p < 0.001, a Nagelkerke R Square of 29.3%, and well-fitting model with the goodness of fit test (p=0.723)). However, among these factors, the presence of cardiac disease (odd ratio (OR) 7.6, 95% confidence interval (CI) 2.71-21.45, p<0.001), anemia (OR 2.5, 95% CI 1.01-6.09, p=0.049), previous syncope (OR 2.5, 95% CI 1.02-6.27, p=0.049, and family history of syncope (OR 10.1, 95% CI 2.08-49.32, p=0.004) emerged as significant independent predictors for syncope (Table 3).

**Table 3:** Predictors of syncope during performing domestic pilgrimage P-value <0.05 is significant

Parameters	Odds ratio (OR)	p-value	95% Confidence Interval for OR	
			Lower	Upper
Age (18-24)	0.135	0.167	0.023	5.371
Age (25-34)	0.178	0.259	0.009	3.557
Age (35-44)	0.377	0.484	0.024	5.807
Age (45-54)	0.145	0.154	0.010	2.059
Age (55-64)	0.309	0.382	0.022	4.304
Age (>65)	0.524	0.614	0.042	6.483
Nationality (Saudi)	0.943	0.861	0.489	1.818
Job (non-employed)	1.240	0.666	0.521	4.931
Job (student)	1.230	0.614	0.550	2.747
Job (employed)	2.298	0.419	0.306	17.263
Gender (males)	0.907	0.782	0.457	1.803
Obesity	1.510	0.251	0.748	3.048
Hypertension	1.347	0.572	0.479	3.792
Diabetes	0.961	0.932	0.386	2.395
Hyperlipidemia	0.988	0.981	0.362	2.696
Cardiac disease	7.6	<0.001	2.71	21.45
Anemia	2.5	0.049	1.01	6.09
Previous syncope	2.5	0.045	1.02	6.27
Family history	10.1	0.004	2.08	49.32
Hypotension	1.736	0.198	0.749	4.024
Constant	0.033	0.031		

## Discussion

The results of the present study provide characteristic data regarding the prevalence, triggers, and predictors of syncope among domestic pilgrims who performed Hajj during one of the hottest summer months in 2023. Results showed an 18.1% prevalence of syncope, sometimes more than once, with reported traumatic injuries in almost half of them. Syncope happened for the first time in 10.31%, with a 2.5-fold increase in the likelihood of occurrence among those with a previous history of syncope. People with recurrent syncope start fainting by age 30, and many clinical studies report syncope recurrences over subsequent decades [7], suggesting a genetic origin for their vasovagal syncope [8]. Pooling family data [9,10] reported that 36-51% of patients who experienced fainting had a positive family history with a pattern compatible with incomplete penetrance of autosomal dominance [11]. This is in accordance with our results, where the strongest predictor of syncope was the positive family history.

In this study, cardiac patients have a 7.6-fold increased likelihood of developing syncope during the pilgrimage. This finding is of utmost importance as there is enough evidence that cardiac patients have increased rates of both mortality and morbidity during the pilgrimage [5]. Syncope of cardiac origin results from compromised cardiac output secondary to either structural, mechanical, or dysrhythmic causes. Anemia among domestic pilgrims increased the likelihood of syncope by 2.5-fold. It is well known that the gradual onset of anemia is associated with compensatory mechanisms that minimize the symptoms. In anemia, due to acute blood loss, the reduction in oxygen-carrying capacity and hypovolemia results in hypotension with a risk of syncope. Hajj situation exposes pilgrims to multiple situations, which can lead to hypovolemia that precipitates symptoms even in patients with chronic anemia.

Triggers of syncope were mainly the combinations of orthostatic intolerance with other aggravating factors like prolonged standing in a stressful crowding. These triggers are common during Hajj rituals, especially on the day of Arafat. Orthostatic intolerance is defined by a sustained reduction of systolic blood pressure of at least 20 mmHg or diastolic blood pressure of 10 mmHg within three minutes of standing [12].

Around 18.8% of cases of syncope didn’t seek medical advice, which may be attributed to the transient nature of loss of consciousness. However, this finding represents a clue to the absence of awareness of pilgrims about the associated risk of syncope, especially among cardiac patients [5]. However, the diagnosis of syncope is often challenging, as the causes are complex and often multifactorial. This is clearly seen in this study as medical consultation provided multiple explanations in 42% of cases with syncope, with heat illness and its associated dehydration as the major possible causes. During mass gatherings, a one-degree increase in temperature could result in an 11% increase in the number of individuals requiring medical attention. Excessive exertion during pilgrimage rituals was the cause in 26.1% of cases [13]. Exercise-associated collapse is seen even among healthy athletes [14], possibly due to exercise-induced postural hypotension. Postexercise systolic blood pressure may drop by 20 mmHg below supine values on assuming the upright posture [15].

In extreme conditions, a combination of heat exposure and exertion may lead to exertional heat stroke, characterized by collapse or syncope associated with hyperthermia [16].

Limitations

This study has some limitations. First, the cross-sectional study design allows recall bias. Second, only domestic pilgrims from KSA were included. Domestic pilgrims are at an advantage compared to external pilgrims as they are accustomed to the weather, habits, and cultures. This could limit the generalization of the results to all pilgrims. However, many faced the risk of syncope, drawing attention to the increased risk among other pilgrims. Third, there are only a few seniors in the study population (1.4%), which again limits the generalization of the results to the elderly populations in whom cardiovascular morbidity plays a more important role in the etiology of syncope [17].

## Conclusions

Syncope during domestic pilgrimage is common, especially on the day of Arafat, and carries the risk of traumatic injury. People with personal or family history of syncope and comorbidities, especially cardiac patients, are prone to this risk. Healthcare should focus on risky patients on days and increase pilgrims' awareness about triggers of syncope, especially sudden and prolonged standing, exertion, and heat exposure, and seeking medical help immediately.

## Appendices

Appendix A

Table 4 shows the pilgrimage data collection sheet of syncope among Hajj domestic pilgrims, 2023.

**Table 4:** Syncope among internal pilgrimage data collection sheet

A- Demographic and clinical data
I-Gender:
Male-1
2-Female
II-Age:
1- under 18 years old
2- 18-24 years old
3- 25-34 years old
4- 35-44 years old
5- 45-54 years old
6- 55-64 years old
7- Above 65 years old
III- What is your occupation:
IV- Do you have obesity
1-Yes
2- No
V- Do you have Hypertension?
1-Yes 2- No
VI-Do you have Diabetes?
1-Yes
2- No
VII-Do you have Hyperlipidemia?
1-Yes
2-No
VIII- Do you have cardiac disease?
1-Yes
2-No
IX- Have you been told that your blood pressure is usually low?
1-Yes
2-No
X- Have you been told that you have anemia?
1-Yes
2-No
XI- Have you performed Hajj before?
1-Yes
2-No
XII- If yes, do you remember which year you performed Hajj?
1-2023-1444
2-Before 2023-1444
XIII- Do you have any other medical conditions?
1-Asthma
2-thyroid disease
3-Anemia
4-autoimmune disease
5-Obesity
6-other
B- History of fainting
I- Have you ever fainted in your life?
1-Yes
2-No
II- If yes, how many times have you fainted?
1-Once
2- 2-5 times
3- 6-10 times
4-More than 10 times
III- what was your condition when you fainted?
1-Standing
2-Sitting
3-Exercising
4-Walking
5-In a crowded place
6-In a stressful situation
7-After standing up quickly
IV- Did anything happen before you fainted, such as feeling lightheaded, nauseous, or dizzy?
1-Yes
2-No
V- Did you lose consciousness completely?
1-Yes
2-No
VI- How long did you stay unconscious?
1- Less than a minute
2- 1-5 minutes
3- 6-10 minutes
4-11-30 minutes
5- More than 30 minutes
6-I don't know
VII- Did you have any injuries when you fainted?
1-Yes
2-No
VIII- Have you ever had any other episodes of dizziness or lightheadedness?
1-Yes
2-No
IX- Are you taking any medications that could be related to syncope?
1-Yes
2-No
X- Did you suffer from stress or emotional distress at the time of syncope?
1-Yes
2-No
XI- Do you have any family history of syncope?
1-Yes
2-No
C: Fainting during performing Hajj
I- Have you ever fainted while performing Hajj?
1-Yes
2-No
II- If yes, how many times have you fainted while performing Hajj?
Once
2-5 times
6-10 times
More than 10 times
III- what was your condition when you fainted while performing Hajj?
1-Standing
2-Sitting
3-Exercising
4-Walking
5-In a crowded place
6-In a stressful situation
IV- Did anything happen before you fainted, such as feeling lightheaded, nauseous, or dizzy while performing Hajj?
1-Yes
2-No
V- Did you lose consciousness completely while performing Hajj?
1-Yes
2-No
VI- How long did you stay unconscious while performing Hajj?
1-Less than a minute
2- 1-5 minutes
3- 6-10 minutes
4-11-30 minutes
5- More than 30 minutes
6-I don't know
VII- Did you have any injuries when you fainted during performing Hajj?
1-Yes
2-No
VIII- At which part of performing Hajj did you faint?
1. Tawaf (circumambulation) around the Kaaba
2-Sa'i (walking between Safa and Marwa)
3-Standing at Arafat
4-Mina rituals
5-Stoning of the Jamarat (throwing pebbles at pillars)
6-During the Tawaf Al-Ifadah (Tawaf of Hajj)
7-During the farewell Tawaf (Tawaf Al-Wida)
IX- Did you seek medical advice?
1-Yes
2-No
X- What was the reason the doctor suggested for fainting?
1-Low blood pressure
2-Dehydration
3-Heat exhaustion
4-Overexertion
5-Low blood sugar
6-Anxiety or stress
XI-Triggers of fainting during performing Hajj?
1- High environmental temperature
2-Prolonged standing
3-Prolonged walking
4-Psychological stress
5-Sudden changes in posture
6-Fasting
7-Consuming food
8- Consuming water
9-Other triggers(mention)
